# Research on Convex Fiber Grating Tactile Sliding Sensor Based on Mechanical Fingers

**DOI:** 10.3390/s24113374

**Published:** 2024-05-24

**Authors:** Guan Lu, Shiwen Fu, Yiming Xu

**Affiliations:** 1 School of Mechanical Engineering, Nantong University, Seyuan Road, No. 9, Nantong 226019, China; luguan@ntu.edu.cn (G.L.); 2109310002@stmail.ntu.edu.cn (S.F.); 2 School of Electrical Engineering, Nantong University, Nantong 226019, China

**Keywords:** touch-sliding sensing, FBG sensor array, mechanical finger, convex type, sliding recognition

## Abstract

In order to solve the problem of flexible sliding tactile composite sensing in the actual grasp of intelligent robot fingers, this paper proposes a research on a convex fiber grating tactile sliding sensor based on mechanical fingers. Based on the sensing principle of fiber Bragg grating, 3D printing technology was used to encapsulate the FBG sensor array with elastic 50 A resin, a double-layer “hemispherical cuboid” distributed sensing unit was designed, and the FBG slippery tactile sensor was actually pasted on the surface of the mechanical finger for static and dynamic experiments. The experimental results show that the slippery tactile sensor designed in this paper has a good linear relationship with temperature and strain. The temperature sensitivities of the polymer-packaged FBGs are $K_{T1}$ = 13.04 pm/°C and $K_{T2}$ = 12.91 pm/°C, and they have a pressure sensitivity of 40.4 pm/N and 31.2 pm/N, respectively. The FBG sliding tactile sensor not only realizes the identification of the sliding signal generation point and the end point but also completes the classification and identification of sandpaper, cardboard, and polypropylene plastic, and it has a high degree of fit with the robot finger, which has certain application value for the intelligent robot sliding tactile signal perception.

## 1. Introduction

The ability of touch perception directly reflects the intelligence level of the bionic body. At present, the research of touch sliding sensing at home and abroad has developed and increasingly tended toward miniaturization, integration, and intelligence. However, due to the weak anti-electromagnetic interference ability, lack of flexibility, and insufficient response speed, various sensors do not meet the real-time and high-precision touch-sliding information detection requirements of bionic bodies, and they have certain limitations.

Fiber Bragg grating has been widely used in various fields in recent years because of its advantages such as small size, high sensitivity, fast response speed, and strong anti-electromagnetic interference ability [1]. Gao et al. designed a triaxial force sensor based on FBG and realized triaxial force detection with a resolution of <1 g [2]. Jiang Qi et al. designed a robot fingertip sliding sensor based on FBG, proposed the second-order derivative algorithm and KNN surface recognition algorithm, and effectively detected and recognized the sliding signal [3]. Feng Yan et al. used the variance of wavelength difference of the three FBG centers and the magnitude and direction of wavelength offset to detect the change of contact force, sliding rate, and direction during the sliding process [4]; Zhou Hui Juan et al. designed and produced a double-sided pressure-sensing earth pressure sensor based on a rhomboid metal frame structure and a metalized fiber grating. The external pressure is amplified through the rhomboid metal structure and acts on both ends of the grating, ensuring high measurement accuracy and the good long-term stability of the sensor [5]; Lu Xingzhi et al. proposed a demodulation method of an FBG sensing system based on nonlinear wavelength drift calibration with an F-P standard to solve the problem of an insufficient demodulation accuracy of the FBG demodulation system due to wavelength drift under variable temperature environments [6]. Long Yuheng et al. proposed a flexible single “cross” and double “cross” touch sliding sensing composite sensor based on FBG that can effectively distinguish sliding signals [7]; Feng Yue et al. designed a soft finger tactile sensing system based on FBG to realize surface texture recognition, reconstruction, and object hardness recognition [8].

To summarize, fiber Bragg grating is becoming more and more widely used in the sensing field, but the research on fiber Bragg grating sliding tactile sensors has mainly focused on the single measurement of a touch signal and sliding signal, and the sliding sensing ability needs to be improved. At the same time, the structure and size of the fiber Bragg grating are large, and there are few sliding tactile sensors that can be actually applied to robot fingers. In the electrical tactile sensor, Xu Decheng, Xin Yi, Huang Ying and other researchers designed a convex sensor with high sensitivity [9,10,11]; however, few people have designed bumps in optically slidingpery tactile sensors, and it is difficult to accurately capture sliding signals by using the plane.

Therefore, in order to achieve sensitization and miniaturization, this paper proposes an FBG “hemisphere”-type sliding tactile sensor based on robot fingers. The hemisphere projection is used to increase the sliding sensing sensitivity of the sensing unit. Here, 3D printing technology is adopted, and elastic 50 A resin is used as the packaging material, which not only protects the fiber grating but also increases the sensitivity of the sensing element with good flexibility and fit degree. The static contact and sliding signals of robot fingers can be detected at the same time, which is of great significance for the research and application of FBG sliding tactile sensors in robot intelligent perception.

## 2. FBG Sensing Principle

The characteristic of FBG sensors is that the periodic refractive index is distributed on the fiber core, and the phase grating is formed in the fiber core by the interaction of germanium ions and external incident photons. According to the coupled mode theory, during the internal transmission of incident light, the fiber Bragg grating will generate coherent reflection on the broadband light in the incident light that meets the relative frequency and incident conditions, forming a central reflection peak; that is, the reflection of incident light must meet the following requirements [12,13]:

(1) $$\lambda_{B}=2n_{eff}\Lambda$$

In the formula, $\lambda_{B}$ is the FBG reflection wavelength, $n_{eff}$ is the effective refractive index of the optical fiber core, and Λ is the grating pitch.

When FBG is affected by changes in temperature and strain, it will also cause wavelength shift, which can be expressed as

(2) $$\frac{\Delta \lambda_{B}}{\lambda_{B}}=\left(1-P_{e}\right)\varepsilon +\left(\alpha +\xi \right)\Delta T$$

In the formula, $\Delta \lambda_{B}$ is the central wavelength drift of the grating, $P_{e}$ is the effective elastic optical coefficient of the fiber material, ε is the axial strain, α is the thermal expansion coefficient of the optical fiber material, ξ is the thermo-optical coefficient of the optical fiber material, and ΔT is the temperature change.

## 3. Sensor Structure Design

### 3.1. Sensor Array Design

**Figure 1:**
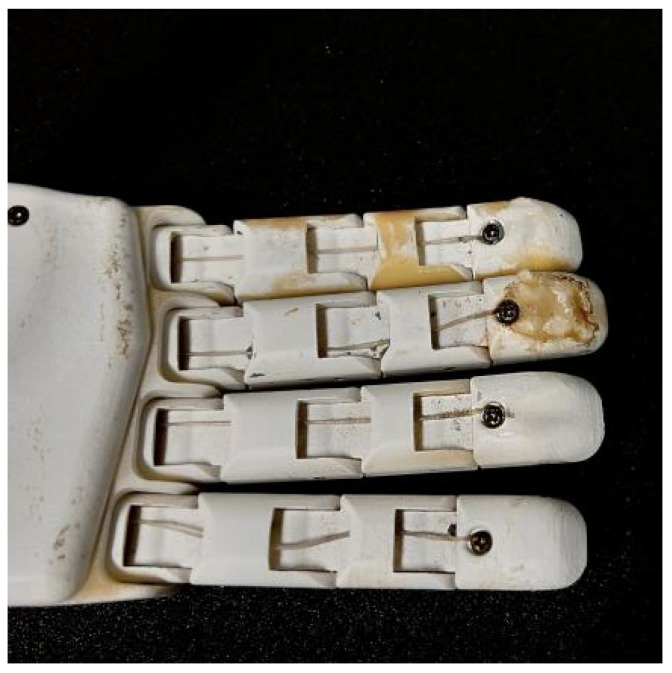
Robot finger.

The robotic finger used in this study, as shown in Figure 1, consists of three phalanges. The inner side of the distal phalanx is selected for the fitting and installation of the tactile sensor. Considering the sensor’s dual-layer layout, the upper hemisphere structure of the tactile sensor is estimated to have a size of approximately R = 3 mm, while the lower rectangular prism measures 7mm×7mm×2mm.

### 3.2. Simulation Analysis of Embedment Depth

**Figure 2:**
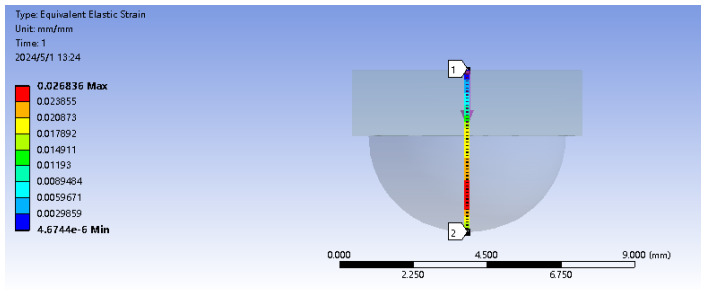
Vertical path under the action of positive force (Vertical path from 1 to 2).

**Figure 3:**
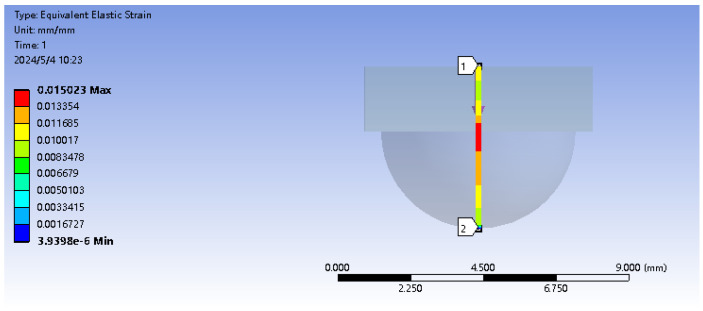
Vertical path under the action of lateral force (Vertical path from 1 to 2).

**Figure 4:**
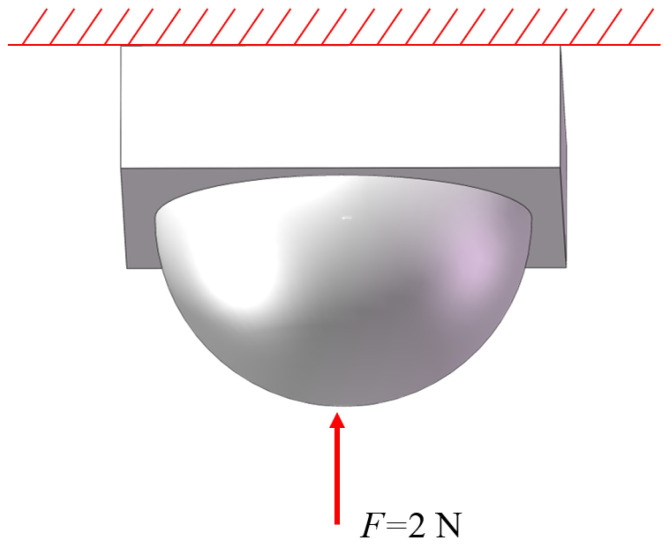
Force position and direction.

Encapsulating fiber Bragg grating sensors with packaging material aims to enhance protection and sensitivity. This approach effectively safeguards the fragile nature of fiber Bragg gratings while also boosting sensor responsiveness. The sensor unit is encapsulated using elastic 50 A resin material with the upper substrate designed in a “hemisphere” structure. Initially, in SolidWorks, the upper hemisphere structure with dimensions of R = 3 mm and the lower rectangular prism measuring 7mm×7mm×2mm are created. The combined elastic body model is imported into ANSYS to conduct finite element analysis using ANSYS Workbench, aiming to determine the optimal embedding depth of the elastic body under loading conditions. Firstly, the elastomeric model is established in Geometry, and in the material data, the density of the matrix model ρ is set at 1000 Kg/m^3^, the elastic modulus E is 9.2 MPa, and the Poisson’s ratio is 0.49. At the center point of the base model, a vertical path is set, while horizontal paths are set at the half of the hemisphere and at the interface between the hemisphere and the lower base. A vertical downward concentrated force of 2 N is applied to the center point of the upper surface of the hemispherical matrix, and a fixed support is added to the lower surface of the cuboid matrix. The simulation results show that the response of the vertical path under the action of vertical force is shown in Figure 2. When 2 N force is applied to the side of the hemisphere, the simulation results show that the response of the vertical path under the action of lateral force, as shown in Figure 3. The stress position and direction of the specific structure are shown in Figure 4.

**Figure 5:**
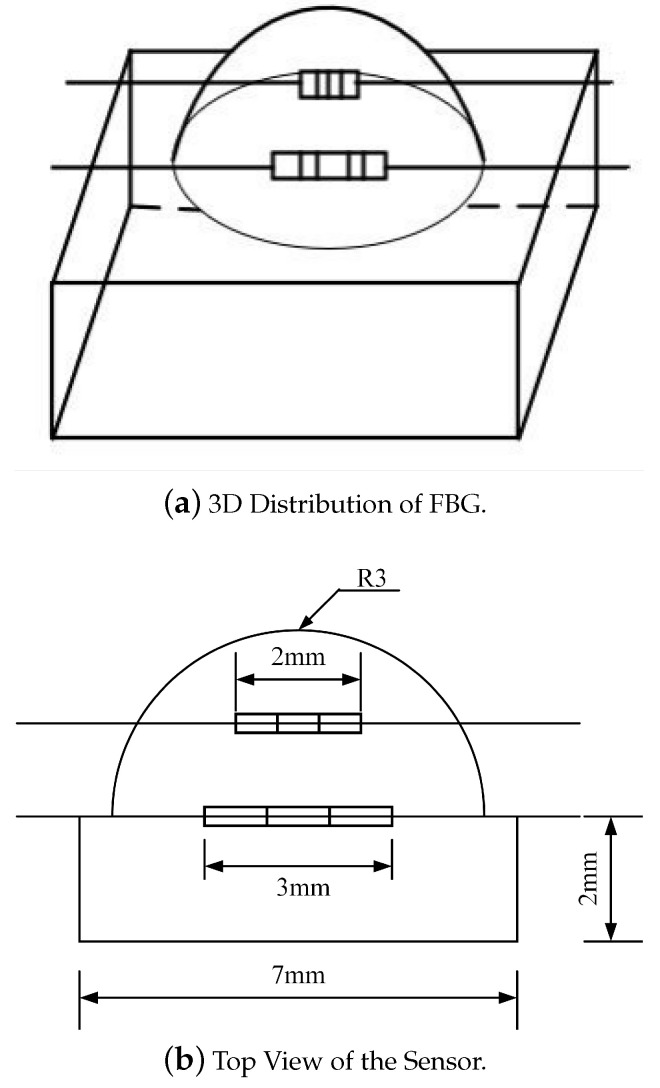
Sensor structure.

From Figure 2, it can be observed that under a force of 2 N, the depth of embedding is 1.5 mm from the vertex of the hemisphere where the overall strain mean value is maximum. From Figure 3, it is evident that under a lateral force of 2 N, the depth of embedding is at the interface between the hemisphere and the cuboid, where the overall strain mean value is maximum. In conclusion, to achieve a real-time and accurate perception of tactile and sliding information from the sensor while avoiding distortion of the grating wavelength due to cross-contact between multiple FBGs, a dual-layer “hemisphere-cuboid” distributed FBG sensing structure is designed with two FBGs embedded separately within the sensor. Based on simulation results, the embedding depths are determined to be halfway into the hemisphere and at the interface between the hemisphere and the cuboid. The sensor structure is illustrated in Figure 5.

**Figure 6:**
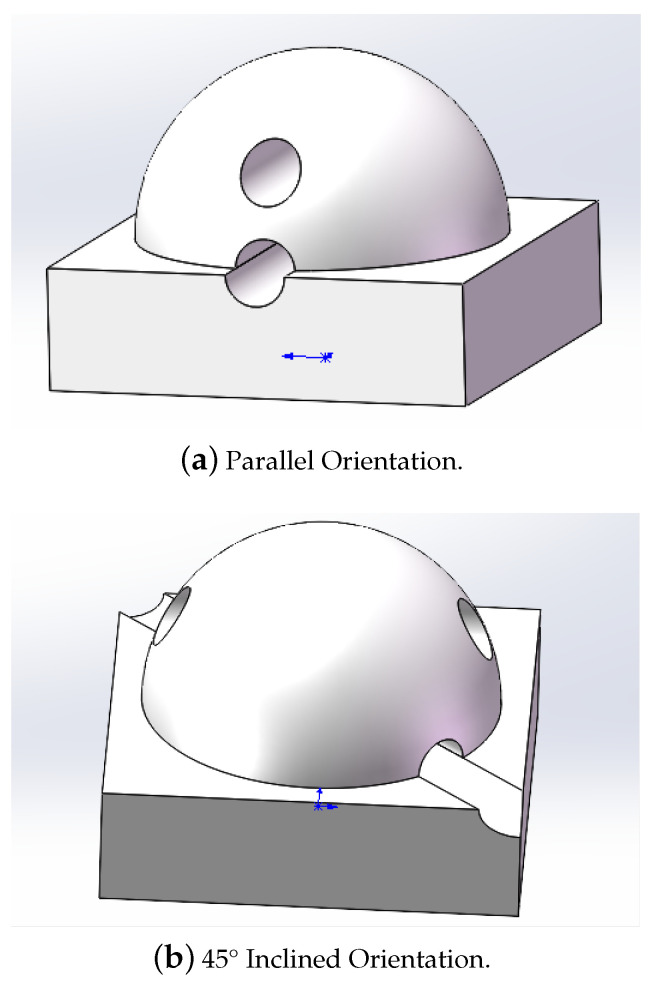
Two types of sensor array structures.

Based on the results of simulation analysis and the optimization of the sensor array structure design, a 2 mm FBG was selected as the sensing element in the upper layer structure with its embedding depth determined to be 1.5 mm from the top of the hemisphere. In the lower layer structure, 2 mm and 3 mm FBGs were chosen as sensing elements with the packaging positions set at 0° and 45° angles inclined from the baseline, which are located at the interface between the hemisphere and the cuboid. The fully packaged fiber optic grating tactile sensor array structure is shown in Figure 6.

## 4. Experiment and Result Analysis

**Figure 7:**
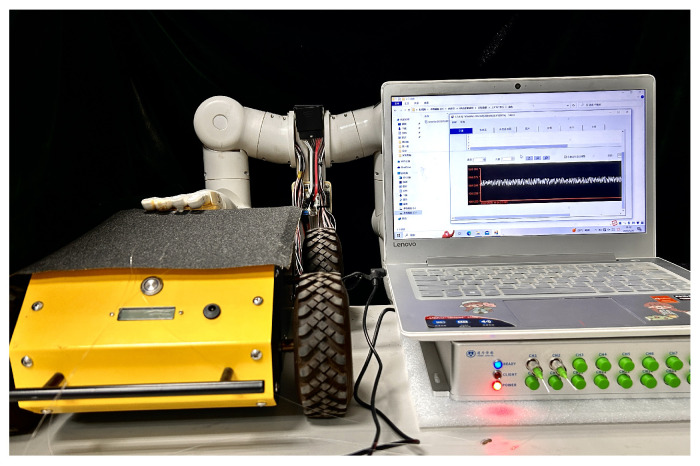
Sliding detection experiment platform.

The dynamic detection platform for the experiment of sliding tactile sensing is composed of demodulation equipment, FBG sensing elements, an intelligent driving car, and a computer. After the sensor is packaged, it is pasted on the surface of the robot fingers. The surface of the car is pasted with different materials such as sandpaper to increase the friction and improve the sensitivity of FBG to the sliding signal. The intelligent driving car moves forward at a constant speed for the sliding experiment. When the object contacts the surface of the elastic body and slides relatively, the center wavelength of FBG will drift correspondingly. The monitoring software in the upper computer can record the change value of the center wavelength in real time. The physical diagram of the detection platform is shown in Figure 7.

### 4.1. Fabrication of Flexible Sensors

This paper designs an FBG “hemisphere”-type sliding tactile sensor based on a robot finger. Elastic 50 A resin is selected as the packaging material. The grating length of the FBG is 2 mm and 3 mm.

Considering the small size of the sensor model designed in this paper, 3D printing technology is selected for ease of processing. The stereolithography apparatus (SLA) is used to ensure the sensitivity of deformation of the photosensitive resin, and to prevent excessive deformation, protect the fiber Bragg grating from breaking. The fiber Bragg grating sliding tactile sensor array is designed with elastic 50 A resin as the base material, and it needs to be pasted on the fingertip surface of the robot finger in practical applications. When the robot finger contacts the outside world through the surface elastic body, if there is a gap between the elastic body and the fingertip surface of the finger, it will affect the finger’s perception of external stress. Therefore, in comparison with other adhesives, this paper selects AB glue as the adhesive between the robot finger and the sensor array.

In this paper, a total of four sensors are produced, and the sensors used are from the same batch. The numbers are shown in Table 1.

**Figure 8:**
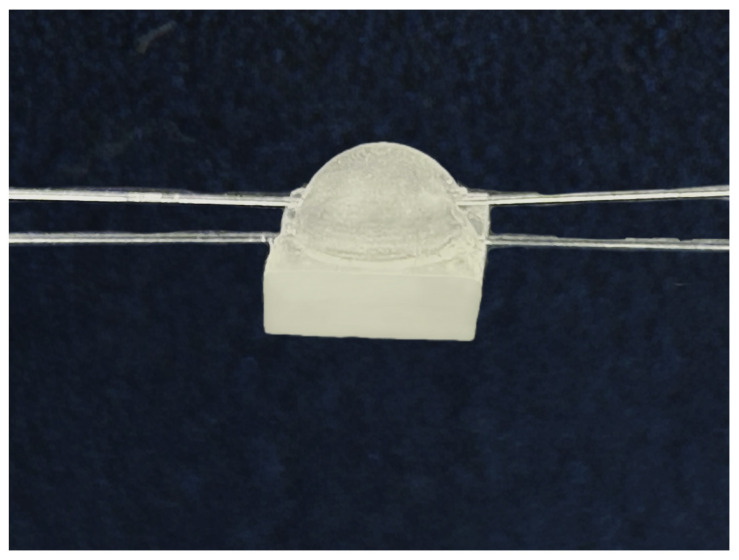
Sliding tactile sensing structure 1.

**Figure 9:**
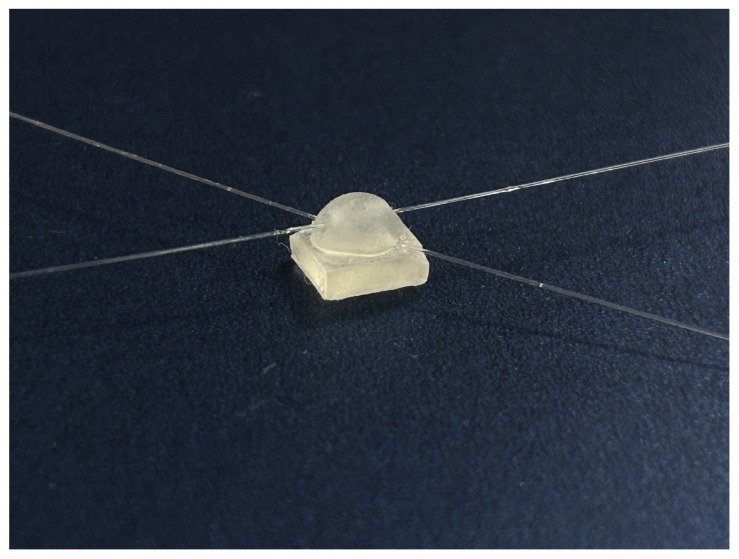
Sliding tactile sensing structure 2.

As an example, the completed sensors using the structure of tactile sensor 1 and tactile sensor 2 are shown in Figure 8 and Figure 9.

The specific technical indicators of the TV1600 Fiber (Tongwei Sensing, Beijing, China) grating demodulation instrument are shown in Table 2 below.

The specific parameters of fiber Bragg grating are shown in Table 3 below.

**Figure 10:**
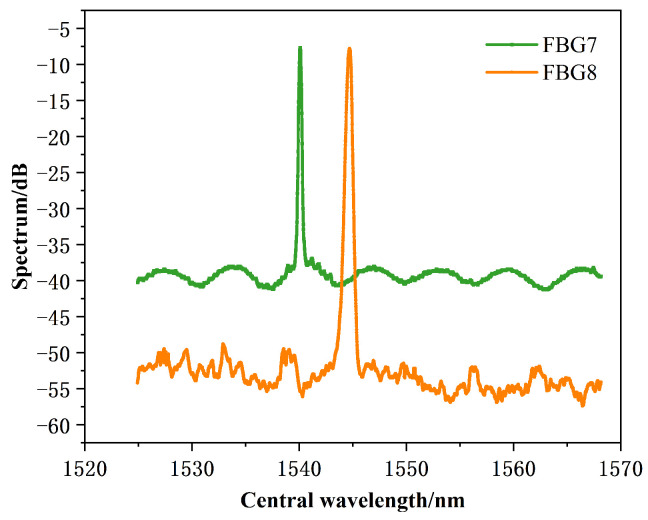
Spectral characteristics.

The spectral characteristics are shown in Figure 10 below.

Although compared to long gratings (e.g., 10 mm), short-length gratings are generally weakly reflective and broadband in the region of maximum reflection. However, it can be seen from Figure 10 that the reflection conditions and measurement resolution can meet the experimental requirements.

### 4.2. Temperature Sensing Experiment and Analysis

**Figure 11:**
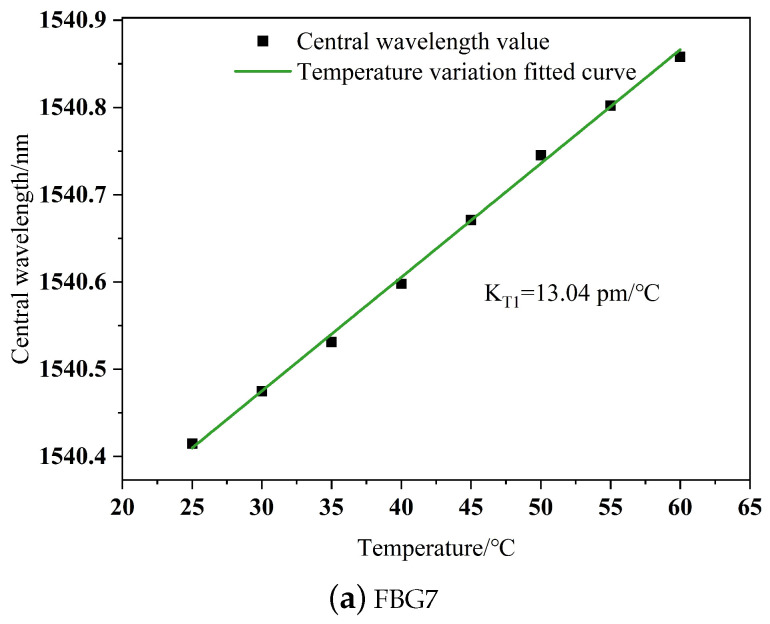

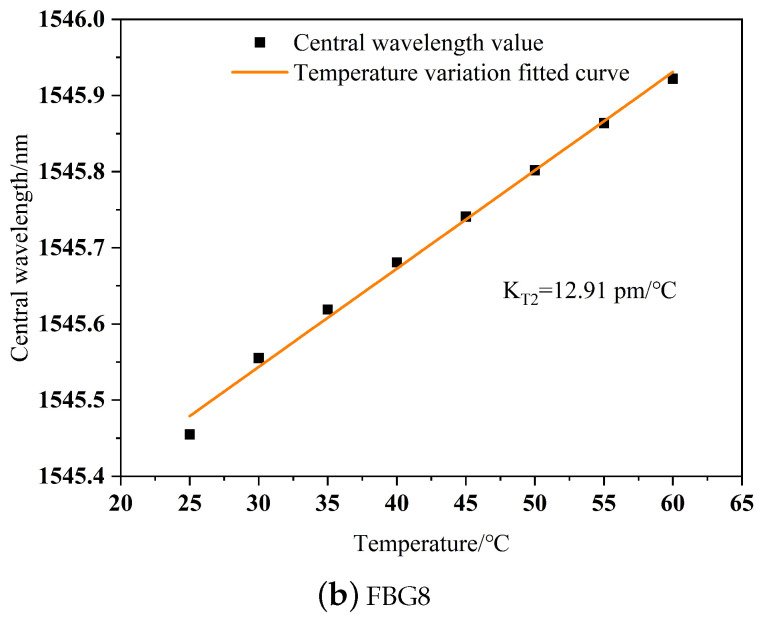
Fitted curve of temperature–wavelength relationship.

Due to the strong sensitivity of fiber Bragg grating sensors to changes in strain and temperature, during the process of a robot finger grasping an external object, the measured strain values may experience a certain degree of deviation due to variations in the external environmental temperature or the temperature of the contacted object itself. In order to investigate the influence of temperature on fiber Bragg grating sensors, temperature-sensing experiments were conducted using a constant temperature box with temperatures set from 25 to 60 °C at intervals of 5 °C on the tactile sensor. Taking sliding tactile sensing structure 4 as an example, the central wavelength drift value was recorded. After numerical analysis, a linear fit was performed, resulting in the temperature-wavelength relationship curve shown in Figure 11.

As shown in Figure 11, the central wavelength of the FBG changes with temperature. The temperature sensitivities of the polymer-packaged FBGs are $K_{T1}$ = 13.04 pm/°C and $K_{T2}$ = 12.91 pm/°C. Due to the effects of packaging and differences in FBG production processes, the temperature sensitivities of each FBG vary slightly, but all maintain good linearity during temperature variations. It is evident that changes in ambient temperature significantly affect the sensing accuracy of the FBG sensors. Therefore, in this experiment, we maintained a constant indoor temperature.

### 4.3. Tactile Sensing Experiment and Analysis

**Figure 12:**
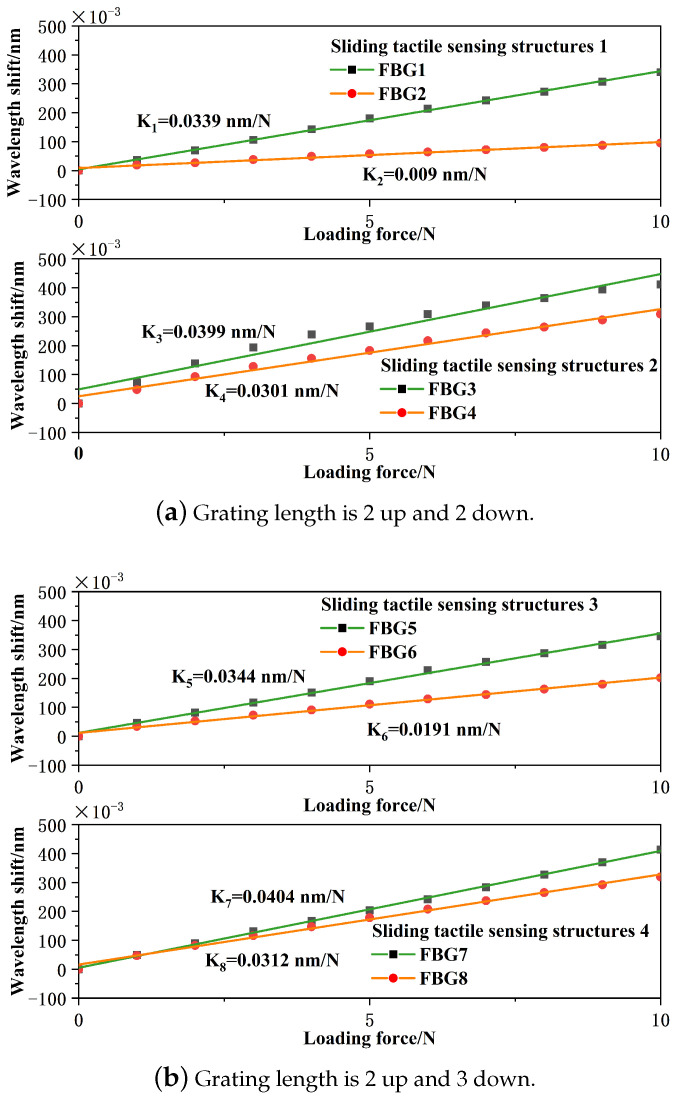
Fitted curves for loading of four sensor structures.

The external pressure was controlled within the range of 0–10 N with a step size set at 1 N. The center wavelength values of each fiber Bragg grating sensor were recorded using the demodulation device’s data acquisition software. Subsequently, Origin software was used to curve-fit the deviations of the center wavelengths of all sensors as a function of applied force during the loading and unloading processes. Taking the loading process as an example, the linear fit curves for the sliding tactile sensing structures 1, sliding tactile sensing structures 2, sliding tactile sensing structures 3, and sliding tactile sensing structure 4 are shown in Figure 12.

From Figure 12, it can be observed that for the dual-layer sensor structures with hemispherical and rectangular shapes incorporating FBG sensors of different lengths, there exists a good linear relationship between the center wavelength of the FBG sensor and the applied force. The overall variation is relatively uniform, and the center wavelengths tend toward a linear distribution, indicating that the FBG sensor possesses good tactile-sensing capabilities. Additionally, based on the linear fit parameters and sensitivities during the loading process as shown in the figure, Table 4 is constructed.

According to the sensitivities and goodness of fit obtained from the hemispherical sensor models with different sensor structures, angles, and different grating lengths in the table, the following can be observed:

(1) Sliding tactile sensing structures 1, 2, 3, and 4 all exhibit good sensitivity and high goodness of fit.
(2) For sliding tactile sensing structures 1 and 2, as well as structures 3 and 4, under the same grating length but different angles, the sensitivities are as follows: FBG3 > FBG1, FBG4 > FBG2, FBG7 > FBG5, and FBG8 > FBG6. It is evident that when using a sensor structure with an upper and lower base inclination angle of 45°, the sensitivity of the sensor is higher.
(3) For sliding tactile sensing structure 1 and 3, as well as structures 2 and 4, under the same angle, comparing different grating lengths shows FBG5 > FBG1, FBG6 > FBG2, FBG7 > FBG3, FBG8 > FBG4. It is evident that when using a 2 mm grating length fiber Bragg grating embedded in the upper layer of the hemispherical rectangular structure and a 3 mm grating length fiber Bragg grating embedded in the lower layer of the hemispherical rectangular structure, the sensitivity of the sensor is higher.

In summary, if considering array structures in the later stages, selecting a sensing structure with 0° orientation both above and below would meet the experimental requirements and facilitate array encapsulation. However, for individual model experimentation, using a 2 mm fiber Bragg grating embedded halfway into the hemisphere and a 3 mm fiber Bragg grating embedded at the junction of the hemisphere and cuboid, along with the 45° inclined angle of the sliding tactile sensing structure 4, yields better results with pressure sensitivities of 40.4 pm/N and 31.2 pm/N, respectively. Furthermore, the fitting coefficients of the upper and lower dual-layer sensors in the sliding tactile sensing structure 4 are 0.999 and 0.995, indicating the excellent linearity and sensitivity of the designed protruding fiber Bragg grating tactile sensor as well as structural stability.

**Figure 13:**
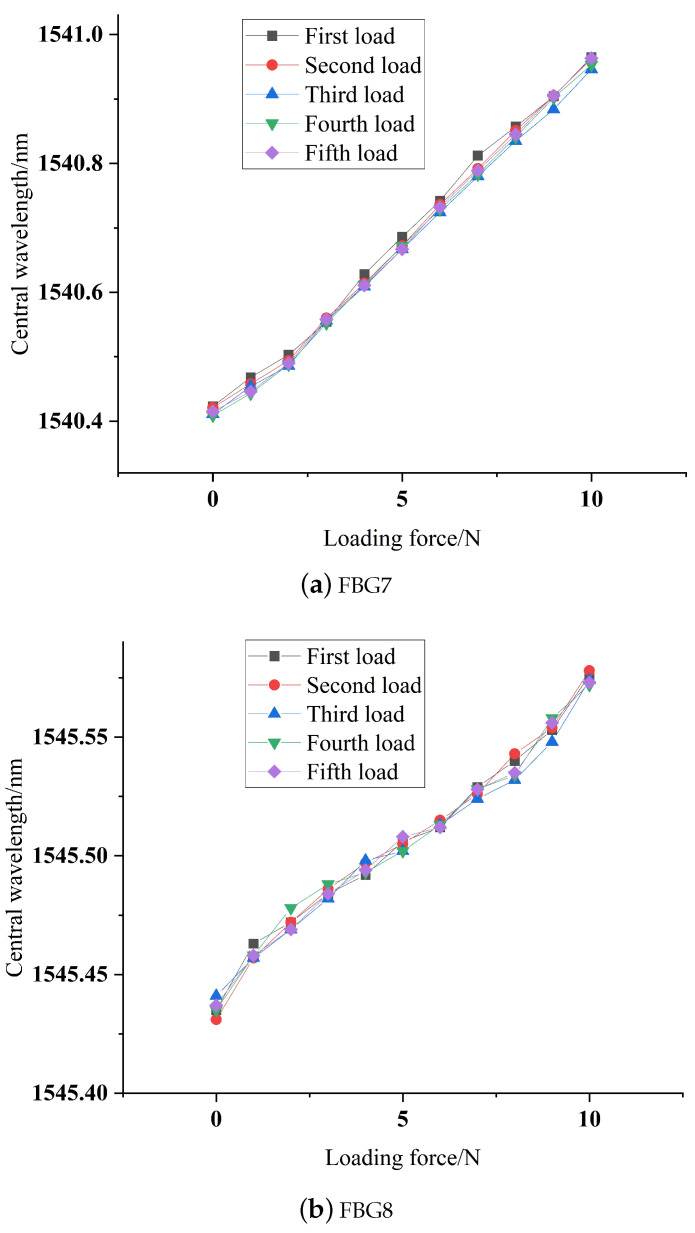
Repeatability fitting curve.

Apply a 0–10 N load to the fabricated array of sliding tactile sensing structure 4, maintain consistent conditions, conduct five repeated experiments, acquire the center wavelength variations of the raised fiber Bragg grating sensors, and illustrate the repeatability curve, as depicted in Figure 13.

It can be observed from the repeatability curve in Figure 13 that the loading curves of the upper and lower two sensors in the sliding tactile sensing structure 4 designed in this paper are highly overlapping, and the overall error is controlled within 5 pm, indicating that the raised FBG slip touch sensor has good repeatability.

**Figure 14:**
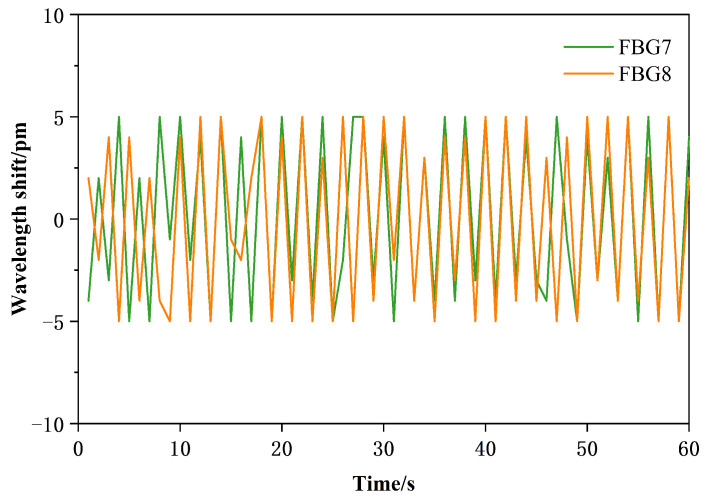
Sensor creep characteristic curve.

To ensure that the temperature of the external environment is constant and unchanged, the completed sliding tactile sensing structure 4 is fixed horizontally at the contact position of the circular end face of the press, and a constant pressure of 2 N is continuously applied to the upper surface of the raised FBG touch-slip sensor array for one minute. During this period, the central wavelength of each sensor above and below the raised FBG touch-slip sensor is recorded, and the creep characteristic curve is drawn, as shown in Figure 14.

From the creep characteristic curve depicted in the above Figure 14, it is evident that under a fixed load condition, the center wavelength variations of the individual FBG sensors in the raised fiber Bragg grating tactile sensor exhibit minimal amplitude changes. The overall center wavelength offset of the sensor array remains within 10 pm, indicating the excellent resistance to creep exhibited by the raised fiber Bragg grating tactile sensor.

### 4.4. Experiment and Analysis of Sliding Sensing

In this experiment, the sliding detection was conducted using the sliding tactile sensing structures 4 with an upper and lower incline angle of 45°. The sliding speed of the object was kept constant, and the contact force applied to the object was also kept constant. Data collected by the demodulator were analyzed using Origin for plotting. The sliding recognition algorithm relied on analyzing the difference in the center wavelengths of FBG7 and FBG8.

**Figure 15:**
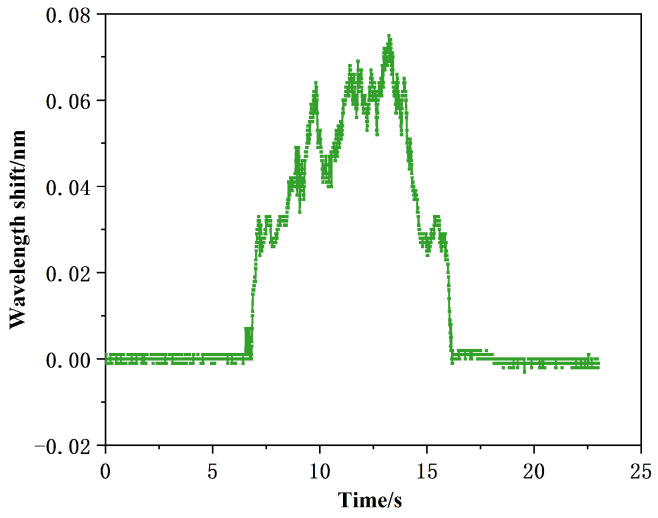
Sliding detection.

The specific implementation method is as follows: Step 1: Collect sliding data for 10–15 s and process the data by subtracting the static pre-sliding data from each data point. Step 2: Set the threshold for sliding recognition as B. When the difference in center wavelengths between FBG7 and FBG8 is consistently greater than the set threshold value multiple times, it is considered the start of sliding. Similarly, after sliding has commenced, if the difference is consistently less than this threshold multiple times, it is considered the end of sliding. Experimental results indicate that the detection performs best when the threshold B is set to 0.02 nm. Figure 15 illustrates the schematic diagram of the sliding detection effect.

**Figure 16:**
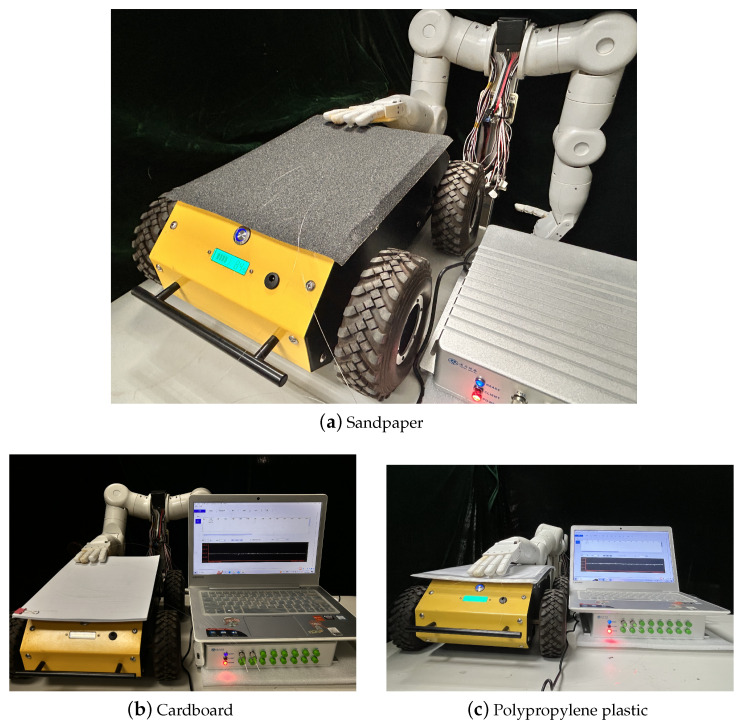
Three different sliding perception materials.

After pasting the sliding tactile sensing structure 4 onto the mechanical finger, a set of samples with different material properties were selected for sliding experiments. The sliding experimental setup is shown in Figure 16, with sandpaper material, cardboard material, and polypropylene plastic material arranged from left to right. An intelligent-driven small car was used to control the sliding speed and direction, and the central wavelength of the fiber Bragg grating was collected during the sliding process for experimental data analysis.

**Figure 17:**
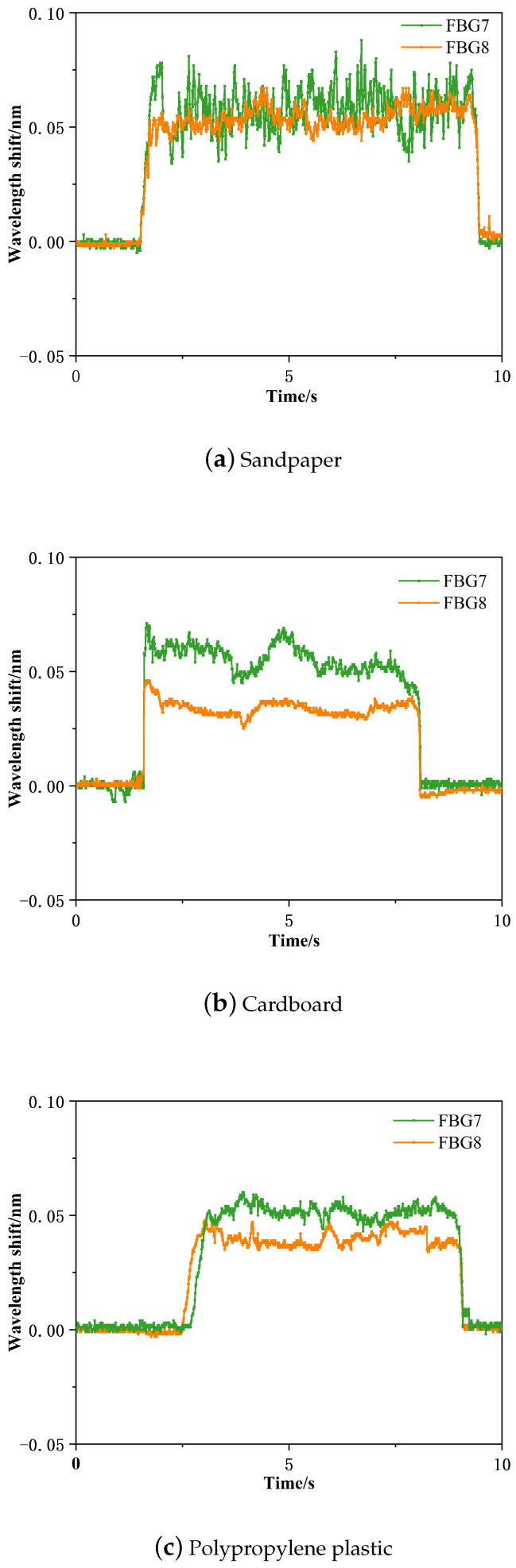
Sliding detection of three different sensing materials.

Three samples with different material properties, namely sandpaper material, cardboard material and polypropylene plastic, were subjected to sliding experiments, and data experiments were conducted to analyze the changes in the central wavelength of fiber Bragg grating during the whole sliding process. Figure 17 shows the schematic diagram of the sliding detection effect under different perceptual materials, including the beginning of sliding, the process of sliding, and the end of sliding.

Figure 17 illustrates the effect of sliding detection with different sensing materials. As the intelligent-driven small car moves, various parts of the polymer elastomer undergo expansion and contraction, causing corresponding changes in the center wavelength of each fiber Bragg grating embedded inside the encapsulation. Figure 17 demonstrates that the distinct changes in the center wavelength of the fiber Bragg grating can clearly distinguish between the stages of pre-sliding, sliding, and post-sliding. Moreover, the friction varies depending on the sensing material in contact during sliding, leading to different behaviors. Consequently, the center wavelength offset of the fiber Bragg grating during the sliding process when in contact with samples of different material properties can vary. In other words, we can determine the surface material properties being sensed by analyzing the signal of the center wavelength offset during the sliding process.

**Figure 18:**
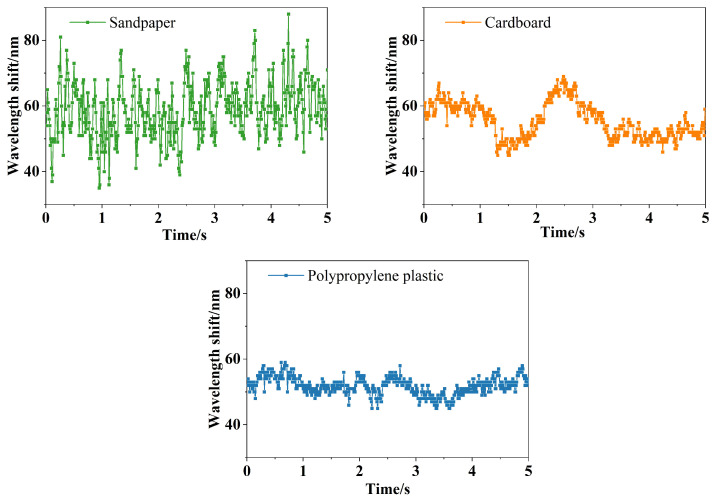
Wavelength shift of three materials.

The wavelength changes in the sliding process under different perceived materials are shown in Figure 18.

Figure 18 shows the center wavelength variation values of the three materials during the sliding process. From Figure 18, it is evident that different materials result in different wavelength variation values during sliding. When in contact and sliding on sandpaper, the center wavelength offset is ±26 pm; for cardboard, the center wavelength offset is ±12 pm; and for polypropylene plastic, the center wavelength offset is ±7 pm. A higher coefficient of friction at the contact interface leads to a larger offset in the center wavelength with more noticeable fluctuations.

**Figure 19:**
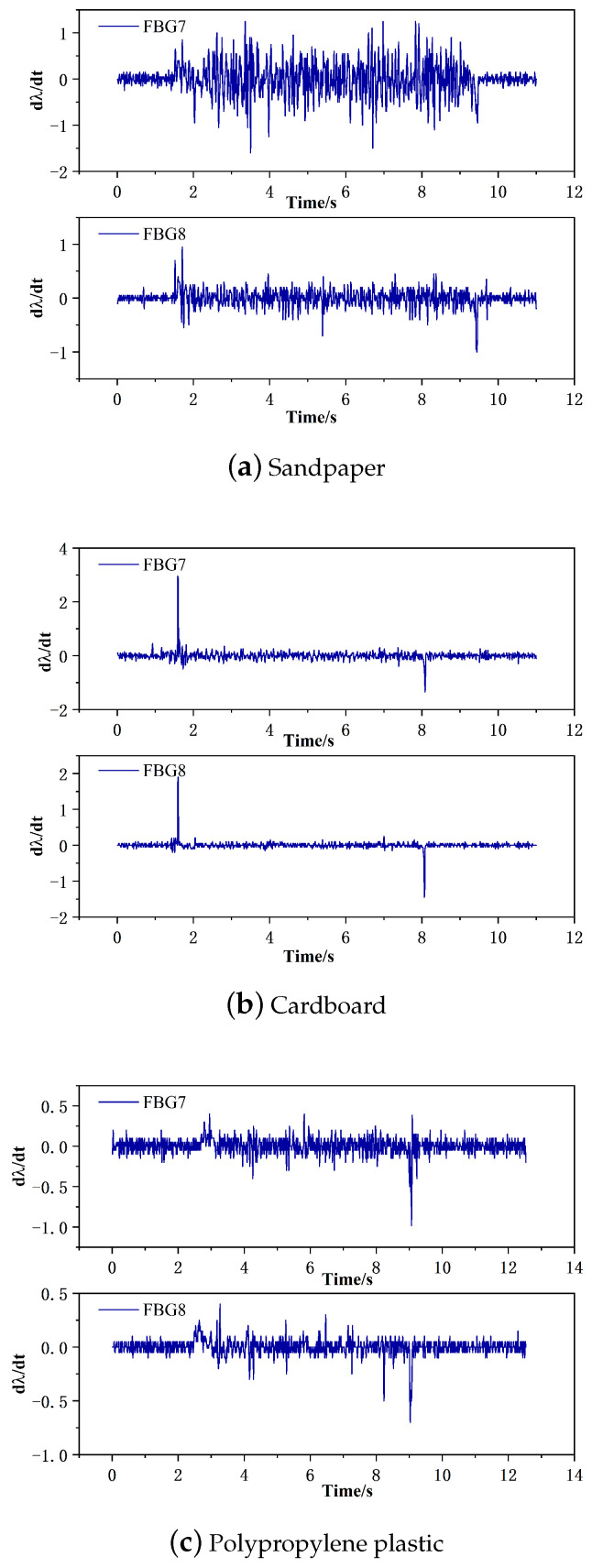
Sliding feature diagram.

When static friction transitions to dynamic friction, there is a sudden change in frictional force. By taking the derivative of the collected wavelength variation data, the points of abrupt change in the first derivative are observed. Figure 19 represents the sliding characteristic graph.

In Figure 19, the blue curve represents the first derivative curve of the wavelength variation. The sudden increase in positive derivative is due to the abrupt change in frictional force caused by the sudden contact between the sliding sensor and the sensing material. The moment when the positive derivative reaches its maximum value and transitions to a negative derivative is caused by the transition from static friction to dynamic friction, resulting in a sudden change in frictional force. The last significant negative derivative change on the curve indicates the moment when the sliding stops. Therefore, analyzing the changes in the derivative can provide insights into the corresponding sliding trends and the start time of sliding. Additionally, based on the above figure, it can be observed that there is good synchronization between the upper and lower layers of the sensor.

## 5. Conclusions

This paper focuses on the research of a protruding fiber Bragg grating tactile sensor based on mechanical fingers. Starting from the sensing mechanism, various sensor structures were designed for simulation and experimental comparison, leading to structure selection and optimization. Ultimately, a “hemisphere-cuboid” dual-layer tactile sensing array was chosen. ANSYS simulation analyses were conducted on different models, and FBG tactile sensing arrays were designed and developed using two methods. The sensors were produced using 3D printing after comparative analysis. An experimental platform was established for the tactile, sliding, and temperature perception of the protruding fiber Bragg grating tactile sensor. The basic performance of the sensor was evaluated. Furthermore, this sensor structure is compact, fits well with mechanical fingers, and during sliding experiments, the FBG tactile sensor was able to identify materials, successfully classifying sandpaper, cardboard, and polypropylene plastic.

(1) The designed convex double-layer touch slip sensor has good linearity, repeatability, and creep resistance performance. The temperature sensitivities of the polymer-packaged FBGs are $K_{T1}$ = 13.04 pm/°C and $K_{T2}$ = 12.91 pm/°C, the pressure sensitivities are 40.4 pm/N and 31.2 pm/N, respectively, the creep change value is controlled within 10 pm, the overall repeatability error is not more than 5 pm, and the structure is miniaturized. It has a high fit with mechanical fingers and later can be made into array model.
(2) A model based on a tactile fiber Bragg grating sensor array with protrusions on mechanical fingertips is proposed. The protrusion-based sensor structure enhances tactile sensitivity on the surface and achieves a sensitizing effect on tactile signals perception. The sensor array is designed as a double-layer embedded structure, utilizing a dual-layer cross structure to enable multidimensional perception, thereby enhancing the stability of the sensor structure and its adaptability to diversity.
(3) A model based on a protruding fiber Bragg grating tactile sensor array with mechanical fingertips was proposed. The protruding sensor structure enhances tactile sensitivity on surfaces, leading to a heightened sensitivity to tactile signals. The sensor array is designed as a dual-layer embedded structure, utilizing a dual-layer cross-structure to enable multidimensional perception, thereby enhancing the stability of the sensor structure and its adaptability to diverse scenarios.

The experimental scope of this study is limited to the tactile slide sensor model, and the intelligent driving car used in the experiment cannot be speed-adjusted; it can only maintain the same constant speed. In the future, we will use a motor slide rail to study the influence of speed on the sliding experiment. In addition, multiple sensor units can be designed to form an array model to detect tactile sliding perception more comprehensively. There is significant flexibility in the arrangement of sensor units. Furthermore, further exploration into the relationship between the surface roughness and the central wavelength of fiber Bragg gratings is warranted. This exploration can advance material identification through the analysis of sliding signals.

## Figures and Tables

**Table 1:** Sensor numbers.

Sensors	Name of FBG	Central Wavelength/nm	Grating Length/mm	Angle
Sliding tactile sensing structure 1	FBG1	1540.329	2	0°
	FBG2	1550.178	2	0°
Sliding tactile sensing structure 2	FBG3	1540.379	2	45°
	FBG4	1550.318	2	45°
Sliding tactile sensing structure 3	FBG5	1540.117	2	0°
	FBG6	1545.301	3	0°
Sliding tactile sensing structure 4	FBG7	1540.431	2	45°
	FBG8	1545.465	3	45°

**Table 2:** Demodulator technical indicators.

Name	Parameter
Number of optical channels	16
Wavelength range	1525 to 1567 nm
Resolution	0.1 pm
Sample frequency	100 HZ
Optical connectors	FC/APC

**Table 3:** Parameters of fiber Bragg grating.

Grid Length/mm	Wavelength Deviation	Reflectivity	3 db Bandwidth	Side-Mode Suppression Ratio	Temperature Range/°C
3	±0.5 nm	>60%	0.65 nm ± 0.2 nm	≥15 dB	−40∼85
2	±0.5 nm	>50%	1.0 nm ± 0.2 nm	≥15 dB	−40∼85

**Table 4:** Fit parameters and sensitivities.

Sensors	Name of FBG	Sensitivity (nm/N)	Goodness of Fit
Sliding tactile sensing structure 1	FBG1	0.0339	0.998
	FBG2	0.0090	0.982
Sliding tactile sensing structure 2	FBG3	0.0399	0.959
	FBG4	0.0301	0.982
Sliding tactile sensing structure 3	FBG5	0.0344	0.996
	FBG6	0.0191	0.994
Sliding tactile sensing structure 4	FBG7	0.0404	0.999
	FBG8	0.0312	0.995

## Data Availability

The data presented in this study are available on request from the corresponding author.
